# Pesticide Residue Screening Using a Novel Artificial Neural Network Combined with a Bioelectric Cellular Biosensor

**DOI:** 10.1155/2013/813519

**Published:** 2013-07-28

**Authors:** Konstantinos P. Ferentinos, Costas P. Yialouris, Petros Blouchos, Georgia Moschopoulou, Spyridon Kintzios

**Affiliations:** ^1^Laboratory of Informatics, School of Food Science, Biotechnology and Development, Agricultural University of Athens, Iera Odos 75, 11855 Athens, Greece; ^2^Laboratory of Enzyme Technology, School of Food Science, Biotechnology and Development, Agricultural University of Athens, Iera Odos 75, 11855 Athens, Greece

## Abstract

We developed a novel artificial neural network (ANN) system able to detect and classify pesticide residues. The novel ANN is coupled, in a customized way, to a cellular biosensor operation based on the bioelectric recognition assay (BERA) and able to simultaneously assay eight samples in three minutes. The novel system was developed using the data (time series) of the electrophysiological responses of three different cultured cell lines against three different pesticide groups (carbamates, pyrethroids, and organophosphates). Using the novel system, we were able to classify correctly the presence of the investigated pesticide groups with an overall success rate of 83.6%. Considering that only 70,000–80,000 samples are annually tested in Europe with current conventional technologies (an extremely minor fraction of the actual screening needs), the system reported in the present study could contribute to a screening system milestone for the future landscape in food safety control.

## 1. Introduction

The contribution of quality tests of exported food and other agricultural commodities to the total food quality sector has a market value of 1.7 billion €. A major part of the initiative for reduced use of pesticides belongs to the food industry and retail trade. In particular, various business-to-business systems have been developed to certify the quality of Integrated Crop Management (ICM) products on a worldwide scale, some of them with considerable success (e.g., EUREPGAP in Europe) [1, 2]. Therefore, there is a vivid demand by the international food producers and industry for pesticide residue screening tools as proximal as possible to the production and processing sites. The issue of screening capacity, realized through rapid, cost-efficient, and high throughput pesticide residue testing, is an indispensable goal, especially considering the astonishingly low number of samples tested annually with conventional methods at certified laboratories all over Europe. The availability of a system providing growers, food companies, and distributors with the flexibility to routinely screen for a range of residues regularly in a cost effective way would allow the identification of remedial solutions quicker than is currently possible. 

As one of currently major cellular biosensor technologies, the bioelectric recognition assay (BERA) utilizes live, functional cells in a gel matrix coupled with a sensor system able to measure changes in the cellular electric properties. Cells that are able to specifically interact with a target analyte produce a unique pattern of electrical potential as a result of their interaction with this analyte.

The BERA working principle has been already utilized for screening pesticide residues as target analytes (more specifically, carbamate and organophosphate pesticide residues in different food matrices [3, 4]). Although this system is sufficient for application by an expert user on a small, laboratory scale, it suffers from a drawback: the inevitable use of an empirical way (examining the biosensor's response data) to identify a pesticide in a sample. It would be highly desirable to avail over a pesticide classification software as a component of the biosensors, at the same time learning during use and therefore obtaining a better classification accuracy.

One option in this direction is the employment of computational models that try to approximate a function from sample data, such as artificial neural networks (ANNs) [5]. Having become popular with the development of the backpropagation training algorithm [6], their training includes a sufficient number of data to “learn” the process behind the production of these data. In the particular case of chemical and biological applications, characterized by highly nonlinear processes, the use of a variety of ANN methodologies has been proven to be very successful [7–10].

In the present study, we developed and trained a customized feedforward ANN [11] for the classification of three different pesticide groups (pyrethroids, carbamates, and organophosphates) detected by a cell-based biosensor operating on the BERA principle and combined with a high throughput measurement device. The novel system classified correctly the presence of the pesticide groups under detection with an overall success rate of 83.6%. The results of the application of the proposed ANN systems support the adoption of the novel classification methodology which can become a key component of an integrated high throughput, rapid, high capacity screening system for pesticide residues.

## 2. Experimental Setup

### 2.1. Materials

Mouse neuroblastoma (N2a), human neuroblastoma (SK-N-SH), and African green monkey kidney (Vero) cell cultures were originally provided from LGC promochem (UK). Standard pesticide solutions were prepared from commercial formulations purchased from various manufacturers (Table 1). Pesticide mixtures were prepared thereof daily in double distilled water. All other reagents were purchased from Fluka (Switzerland). Cells were cultured in Dulbecco's medium with 10% fetal bovine serum (FBS), 1 U *μ*g^−1^ antibiotics (penicillin/streptomycin), and 2 mM L-glutamine. Cells were detached from the culture and concentrated by centrifugation (2 min, 1200 rpm, 25°C), at a density of  2.5 × 10^6^ mL^−1^. During each assay (see below, Section 2.2) cells were used at a density of 1000 *μ*L^−1^.

### 2.2. Biosensor Principle

The biosensor was based on cellular biorecognition elements, which are natural targets of the pesticide groups under investigation. The first two cell lines (N2a, SK-N-SH), being neuronal, are natural targets of all three pesticide groups, due to the inhibition of either acetylcholine esterase (AChE) (organophosphates, carbamates) or ion channels (pyrethroids). Under control conditions (no pesticides present), when acetylcholine is added to the cells, it causes a temporary depolarization of the cell membrane (excitation), which is rapidly cancelled out by the specific cellular mechanisms. However, when pesticides are present, they inhibit these mechanisms (such as AChE), thus allowing for a continuous stimulation of the neural cells. This means that, when AChE/ion channels are inhibited by pesticide residues in the sample, addition of ACh will cause the excessive stimulation of N2a or SK-N-SH cells, which will further lead to membrane depolarization above a predetermined threshold [12].

As documented in previous studies [13, 14], the third cell line (Vero) also responds to the pesticides with a general toxicity response, which is acetylcholine-independent (i.e., no addition of acetylcholine is required).

Following an initial calibration of the biosensor system (analytical results are not shown here), the N2a, SK-N-SH, and Vero cell lines were identified as the optimal biorecognition elements for the pyrethroid, organophosphate, and carbamate pesticide groups, respectively.

### 2.3. Biosensor Device

**Figure 1:**
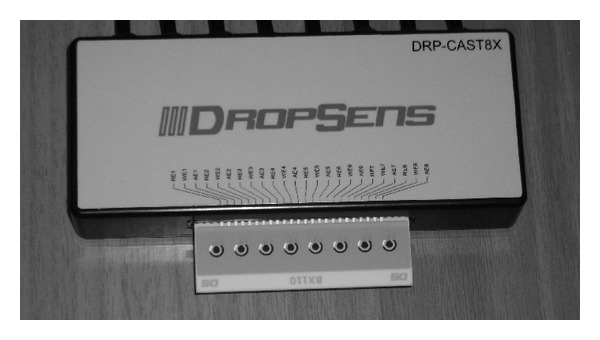
Details of biosensor device used for measuring cell-pesticide interactions. The eight-channel disposable sensor is connected to the potentiometer through a customized interface.

A customized device was developed (Uniscan, Buxton, UK) in order to measure electric signals from the cellular biorecognition elements and allowing for high throughput screening and high speed of assay. The device is a portable potentiometer, having a replaceable guide bearing eight pairs of electrodes connecting on the underside. The system provides a connection interface to insert electrode strips directly into the instrument, utilizing one electrode strip per channel. Each electrode strip comprised a 0.5 mm thick ceramic substrate with three screen printed electrodes (working electrode—WE, reference electrode—RE, and counter electrode—CE). In order to facilitate high throughput screening, DRP-8X110 disposable sensor strips (WE: carbon, RE: Ag/AgCl) bearing eight electrode pairs (corresponding to eight measurement channels) were purchased from DropSens (Asturias, Spain) (Figure 1). Thus, the potentiometer, through its array of eight electrode pairs, received measurements from corresponding eight units of cellular biorecognition elements interacting with the assayed sample(s).

### 2.4. Creation of Pesticide Group Mixtures

The next step was to select the pesticides which composed the representative mixtures for each separate pesticide group. The basic criteria for the selection were the occurrence of residues of the respective pesticides as well as their commercial availability. After an extensive survey, we concluded the formulation presented in Table 1. This formulation is constructed in such way to include, within each group, pesticidal compounds which are representative (i) of actual compounds currently used in European agriculture and (ii) of different levels of solubility in water or polar solvents, since nonpolar solvents are not suitable for use with cellular biorecognition elements.

Within each pesticide group, and due to the fact that individual pesticides are associated with different maximum residue level (MRL) values, also depending on the food commodity under investigation, we decided to create the three different mixtures (corresponding to the three different pesticide groups) by adding pesticides at the concentration corresponding to the lowest MRL within each group. In this way, we secured that the biosensor would be developed on the principle of maximum sensitivity, in respect of the cumulative MRL of each group (actually 0.01 ppm for all groups).

Next, we created two types of sample sets (collections) for training the ANN. The first type of sample set was considered the control sample set, which contained pesticide-free samples as well as samples with pesticides at a cumulative concentration of MRL/20 and MRL/10 (i.e., too low). The second type of sample set was the positive sample set, which contained samples with pesticides at a cumulative concentration of MRL/2 and MRL. The relative composition of each MRL dilution in each sample set is presented in Table 2.

### 2.5. Assay Procedure

**Figure 2:**
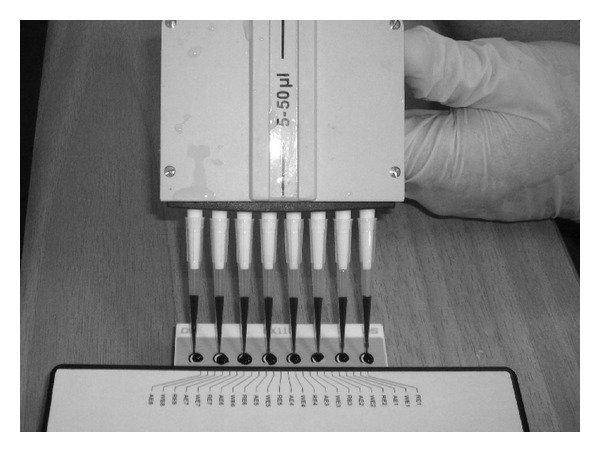
Pouring of cultured cells in suspension placed on the top of each of the eight carbon screen-printed electrodes contained in each disposable sensor strip.

For screening the presence of a particular pesticide group, the corresponding cultured cells in suspension (see Section 2.2) were placed on the top of each of the eight carbon screen-printed electrodes contained in each disposable sensor strip (40 *μ*L *≈* 40 × 10^3^ cells) with the help of a multichannel automatic pipette (Figure 2). Next, the sample was added (pesticide mixture) (5 *μ*L), followed by 5 *μ*L Ach (10 mM).

The response of the cells to the different samples (control and positive sample sets from pesticide group) was recorded as a time series of potentiometric measurements (in Volts). The duration of each measurement was 180 sec, and 360 values/sample were recorded at a sampling rate of 2 Hz.

### 2.6. ANN Design and Training Process

Three main aspects of feedforward ANN modeling were considered for the development of the classification systems:

- network architecture (number of nodes in one or two hidden layers);
- the type of backpropagation training algorithm:
  1. steepest-descent algorithm,
  2. quasi-Newton algorithm,
  3. Levenberg-Marquardt algorithm,
  4. Conjugate-gradient algorithm;
- the type of activation function in the hidden nodes:
  1. logistic function,
  2. hyperbolic tangent (tanh) function.

Trial-and-error experimentations were conducted for the discovery of the best combinations of these parameters. Network weights were randomly initialized, thus several training trials were performed for each possible combination. The widely used methodology of cross-validation [11] was used for terminating the training process, so that over-training was avoided, and good generalization capabilities were ensured. All training algorithms, ANN modeling and experimentations were implemented in MATLAB.

In addition to the trial-and-error approach for the design and parameterization of the ANN model, an evolutionary methodology that combines network design and parameterization with feature extraction from time series was also used (a detailed description of the system is presented in [15]). Its primary goal was to produce meta-data from the information contained in the original time-series, reduce the dimensionality of the input data space, and reduce the noise contained in the initial raw information. A genetic algorithm was used to normalize the initial information and discover the optimal design and parametrization of the ANN model. This evolutionary approach gave poorer results than the trial-and-error approach combined with the feature extraction technique presented.

#### 2.6.1. Meta-Data Creation

Each time series of data consisted of 360 sequential measurements (see also Section 2.5). One way to feed time-series data into an ANN is to convert the information of the time-series data into more suitable meta-data. These meta-data must be much fewer than the number of data samples of each time-series and must capture, in the maximum possible degree, the characteristics of the time series data samples. After some experimentations with several statistical variables (e.g., minimum, maximum, mean, median, standard deviation, skewness) and ways of segmenting the time-series data, from each time series the following set of meta-data was extracted to be used as ANN inputs:

- mean and standard deviation of all 360 data samples;
- mean and standard deviation of each quarter part of the data samples (data samples were divided into four equal-length segments);
- minimum and maximum values of all data samples.

Thus, each time series of 360 measurements was converted into 12 single values.

#### 2.6.2. Initial ANN System Development

As a first approach, we used only the 12 meta-data values as inputs of the ANN model. This approach was initially tested on the development of a classification model for the pesticides of the pyrethroid group. The available data were 450 time series (each containing 360 measurements), that is, 214 control time series (negative) and 236 MRL time series (positive). For each time series, the only available information was the existence or not of pesticide in the sample. These available time series were divided into training and testing sets. 30 random “control” time-series and 30 random “MRL” time series (i.e., 60 in total) constituted the testing set, leaving the rest 390 time-series to form the training set.

The ANN had one output, denoting the existence (value equal to 1) or not (value equal to 0) of the pesticides under question. The training was performed with the values 0 and 1, but the actual output of the network was a real value (normally, but necessarily, between the values of 0 and 1). During the testing of model, all values less than 0.5 were considered to be 0, and all values greater than or equal to 0.5 were considered to be 1.

Several trial-and-error experimentations were conducted, concerning the parameters mentioned before (network architecture, type of backpropagation algorithm, and type of activation functions). The best performance during these training experimentations was given by a 1-hidden-layer network with 10 hidden nodes and hyperbolic tangent activation functions, trained with the quasi-Newton minimization algorithm. 

The ANN that gave the best results during the training and parameterization process described above was further trained for a larger number of training iterations. The performance of the final ANN system was evaluated on the testing data set. The correct classification rate was 70%.

#### 2.6.3. Final ANN Systems Development

The correct classification rate of 70% was not considered satisfactory. Therefore, it was decided that additional information for each time series was necessary. For that reason, two additional parameters were recorded:

- the *age* of the cells (in days),
- the *generation* number of the cells (four different generation values).

These two additional inputs were added to the 12 meta-data inputs, so the final ANNs had in total 14 inputs.

## 3. Results

Following appropriate validation, the ANN architectures and minimization algorithms combinations that gave the best results during training were further trained and tuned, leading to the development of the final ANNs. These models were evaluated in specific testing data sets, that is, new data, different than those used during training. The analytical results for each pesticide group are presented.

### 3.1. ANN Models for the Pyrethroid Group

The available data were 809 time-series (each containing 360 measurements and the corresponding values of the two additional inputs). Specifically, they included 405 control time series (negative) and 404 positive time series. These available time series were divided into training and testing sets. Thirty random “control” time series and 30 random “positive” time series (i.e., 60 in total) constituted the testing set, leaving the rest 749 time series to form the training set.

Similarly to the initial models, the ANN had one output with values corresponding to the existence (1) or nonexistence (0) of pesticides of the pyrethroid group. 

**Figure 3:**
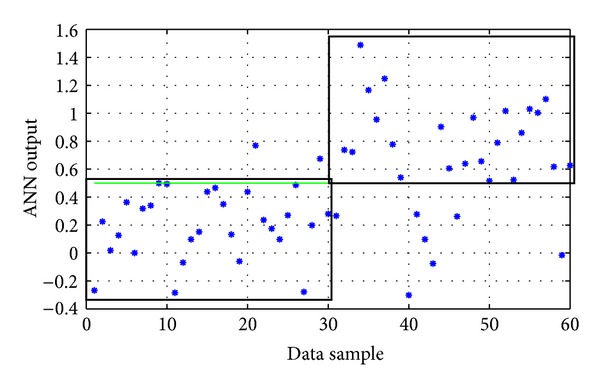
The actual output of *ANN-P1* model on the testing data of the pyrethroid group (the two black boxes represent the correct classification areas). Success rate: 85%.

**Figure 4:**
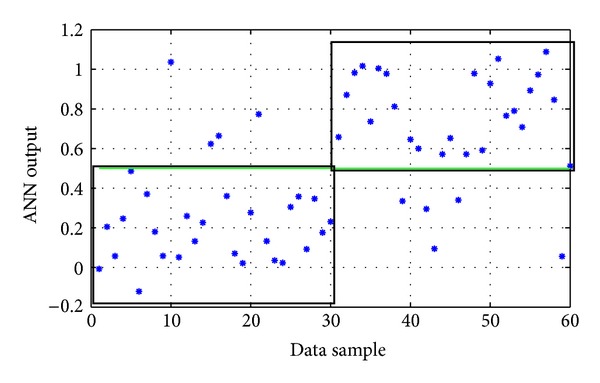
The actual output of *ANN-P2* model on the testing data of the pyrethroid group (the two black boxes represent the correct classification areas). Success rate: 85%.

**Figure 5:**
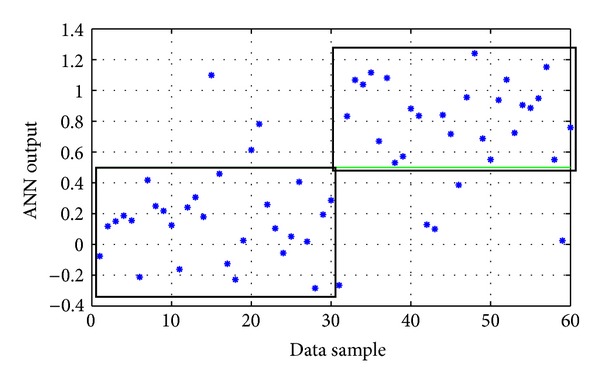
The actual output of *ANN-P3* model on the testing data of the pyrethroid group (the two black boxes represent the correct classification areas). Success rate: 86.7%.

Again, several trial-and-error experimentations were conducted, concerning the usual parameters described before (network architecture, type of backpropagation algorithm, and type of activation functions). The parameters of the ANNs with the best performance during these training experimentations are presented in Table 3, while their corresponding performances on the testing data set are presented in Table 4. Their actual outputs on the testing set are shown in Figures 3, 4, and 5. The best ANN (*ANN-P3*) achieved an overall success rate equal to 86.7%. In comparison, the best ANN designed by the evolutionary approach described in Section 2.6 gave an overall success rate equal to 83%.

### 3.2. ANN Models for the Organophosphate Group

The available data were 1206 time series (each containing 360 measurements and the corresponding values of the two additional inputs). Specifically, they included 506 control time series (negative) and 700 positive time series. These available time series were divided into training and testing sets. Fifty random “control” time series and 50 random “positive” time series (i.e., 100 in total) constituted the testing set, leaving the rest 1106 time-series to form the training set. The ANN had one output with values corresponding to the existence (1) or nonexistence (0) of pesticides of the organophosphates group.

**Figure 6:**
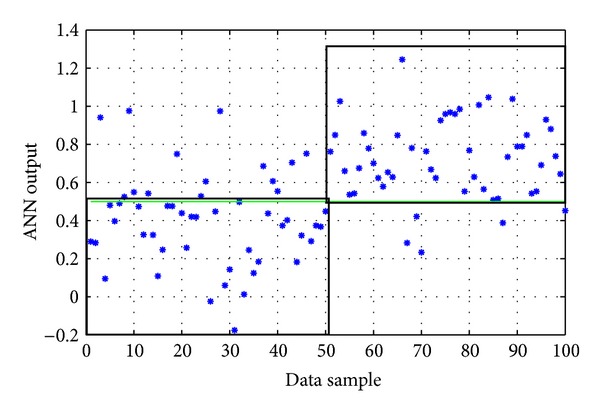
The actual output of the “2-HL/4, 19 nodes/logistic fun's” ANN model on the testing data of the organophosphates group (the two black boxes represent the correct classification areas). Success rate: 81%.

The ANN with the best performance during these training experimentations was a 2-hidden-layer network with 4 and 19 nodes, respectively, and logistic activation functions (linear summation function in the output node), trained with the *Levenberg-Marquardt* backpropagation algorithm. Its evaluation on the testing data set gave a correct classification rate equal to 81% (Table 5). The actual output of the ANN on the testing set is shown in Figure 6. In comparison, the evolutionary-designed ANN gave a correct rate equal to 77%.

### 3.3. ANN Models for the Carbamate Group

The available data contained 1170 time series (each containing 360 measurements and the corresponding values of the two additional inputs). Specifically, they included 585 control time series (negative) and 585 positive time series. These available time series were divided into training and testing sets. Thirty random “control” time series and 30 random “positive” time series (i.e., 60 in total) constituted the testing set, leaving the rest 1110 time series to form the training set. Similarly to the other models, the ANN had one output with values corresponding to the existence (1) or nonexistence (0) of pesticides of the carbamates group.

**Figure 7:**
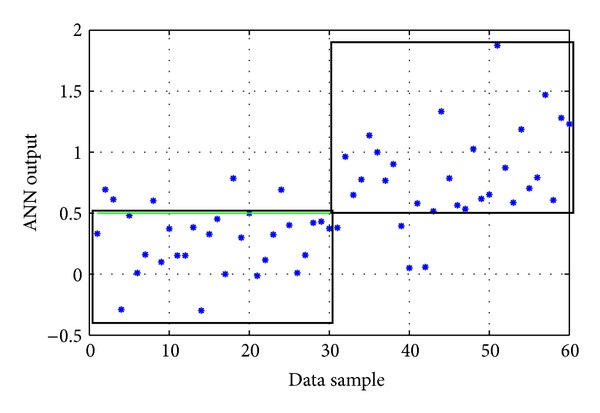
The actual output of the “1-HL/10 nodes/logistic fun's” ANN model on the testing data of the carbamates group (the two black boxes represent the correct classification areas). Success rate: 85%.

The ANN with the best performance during these training experimentations was an *1-hidden-layer network* with 10 nodes and logistic activation functions (linear summation function in the output node), trained with the *Levenberg-Marquardt* backpropagation algorithm. Its evaluation on the testing data set gave a correct classification rate equal to 85% (Table 6). The actual output of the ANN on the testing set is shown in Figure 7. In comparison, the evolutionary-designed ANN gave a correct rate equal to 78%.

## 4. Discussion

Biosensors designed for performing food quality and toxicity analysis can have a significant social, economical and commercial impact. Such sensing units can be of invaluable use for both public authorities (such as custom offices) or private bodies (e.g., food production industry) for the “in situ” monitoring of food quality. Such units will provide reliable information on the food quality, eliminating dangers emerging from adulteration, chemical or biological contamination, improper storage conditions, and chemical residues. 

During the last years, several applications of multiclassifier systems have been developed. These have been particularly useful for the interpretation of data retrieved from biosensors, which are usually associated with difficult pattern recognition problems. For example, a multinet biosensor system for the detection of plant viruses was reported by Frossyniotis et al. [16]. Similar to the present study, the system was based on a BERA sensor. The results showed that the ANN approach performed better than empirical techniques. Another critical parameter in real-life applications, the corresponding time of the proposed classification system, was very competitive compared to the relatively long time required by an expert to make a decision by examining a data curve. Concerning the same end application, Glezakos et al. [17] used the evolutionary approach (see [15] for details) to produce meta-data from the information contained in the original time series, reduce the dimensionality of the input space and drastically decrease the noise contained in the initial raw information.

Over the last years, the use of ANN methodologies in combination with biosensor-based analytical methods is steadily increasing. Typical examples are ANNS used for the detection of glucose and sucrose [18], phenolic compounds [19–21], and neuroactive compounds [22]. In direct association with the present study, ANNs have been used in the biosensor-based determination of various insecticides, such as paraoxon (organophosphate) and carbofuran (carbamate) [23], as well as a series of organophosphate pesticides such as chlorpyrifos-oxon, chlorfenvinphos and azinphos-methyl oxon [24–26], dipterex, dichlorvos, and omethoate [27]. In this context, the novel system presented here allows, for the first time, the successful discrimination among pesticides belonging to three different groups. In this way, it allows for a broader coverage of screened chemical residues than previously achieved. More importantly, perhaps, the novel ANN completes an advanced pesticide screening system in such way that it is fully operational and ready to use. The integrated biosensor platform described in this study has the additional advantage of rapid results (three minutes from sample input to classification report), which makes it attractive for routine use.

## 5. Conclusions

The present report is a demonstration of the classification properties of artificial neural networks, such that they can fully replace the traditional technique of empirical examination of biosensor's response data curve and therefore boosting the utilization potential of the coupled cellular bioelectric assay system. At this stage of development, the performance of the system is quite satisfactory, considering the noisy nature of the measurements and the biological factors involved in the entire process. It was shown that although the initial ANN system performed quite poorly it was radically improved by the inclusion of additional biological factors (age of the cells and their generation number). Obviously, the system can be further improved, but the improvement of the assay principle that would lead to better and more accurate measurements is necessary. The basic scope of the current work was to validate, with real measurements, the viability of the proposed system at different matrix environments. 

The reliability of the novel system is safeguarded due to the use of different cell lines as biorecognition elements, all of which are targets of the screen pesticide groups, as well as the large number of time series used for training. The spectrum of detected substances can be increased by adding other cell lines with differential susceptibility to pesticides. In addition, we envisage that, either by enriching the composition of test (control and positive) sample sets with more pesticide compounds or by creating sample sets for other pesticide groups and then proceed with ANN training, end-users will be able to use the integrated biosensor system for screening essentially all types of residues in food commodities. This is particularly important considering that currently only 70,000–80,000 samples are annually tested in Europe, an extremely minor fraction of the actual screening needs. Therefore, we feel that the present report could contribute to a screening system milestone for the future landscape in food safety control. We currently conduct a series of tests with real samples in order to validate the performance of the system at different food matrix environments.

## Figures and Tables

**Table 1:** Composition of the different pesticide groups used for training the novel ANN (all pesticides were added at a concentration of 0.01 ppm).

Group	Target compounds	Concentration of active compound (%) in formulation	Commercial name	Manufacturer
Organophosphates	Acephate	75	Forten	VETERIN
	Azinphos methyl	25	Azin	AGROCHIMIKI
	Chlorpyrifos	48	Echo	MAKHTESHIM
	Chlorpyrifos methyl	22.5	Reldan	DOW
	Dimethoate	40	Perfekthion	BASF
	Malathion	50	Malathion	AVENTIS
	Methamidophos	50	Tabamor	AGROELLINIKI
	Pirimiphos methyl	50	Actellic	SYGENTA
	Profenofos	50	Selecron	SYGENTA
	Triazophos	42	Hostathion	AVENTIS

Carbamates	Carbendazim	25	Carbendazim	BAYER
	Carbofuran	10	Carbofuran	NITROFARM
	Phenmedipham + desmedipham	8 8	Record	ALFA
	Methiocarb	50	Mesurol	BAYER
	Methomyl	90	Dimethilin	K&N
	Oxamyl	10	Judo	FARMA-CHEM
	Iprodione	50	Rovral Aquaflo	BASF
	Propamocarb	53	Previcur Energy	BAYER
	Thiophanate methyl	70	Neotopsin	K&N

Pyrethroids	Abamectin	1.8	Rotam	ACARAMIC
	Cyfluthrin	5	Baythroid	DU PONT
	Cyhalothrin-lambda	10	Cyhalothrin	SYGENTA
	Cypermethrin	10	Assist	CERDE
	Deltamethrin	2.5	K-Othine	BAYER
	Fenpropathrin	10	Danitol	SUMITOMO
	Fenvalerate	30	Sumicidin	SUMITOMO
	Tau-fluvalinate	24	Mavrik	MAKHTESHIM

**Table 2:** Composition of control and positive sample sets.

MRL dilution	Control sample set	Positive sample set
0	60%	
MRL/20	20%	
MRL/10	20%	
MRL/2		50%
MRL		50%

**Table 3:** Best ANN models for the pyrethroid group.

	Hidden layers/nodes	Activation functions (hidden nodes/output node)	Minimization algorithm in backpropagation algorithm
*ANN-P1 *	1-HL/23	Logistic/linear summation	Levenberg-Marquardt
*ANN-P2 *	2-HL/5, 15	Logistic/linear summation	Levenberg-Marquardt
*ANN-P3 *	2-HL/10, 10	Logistic/linear summation	Levenberg-Marquardt

**Table 4:** Correct classifications for the pyrethroid group (number of samples and corresponding percentages) during the testing process of the ANNs.

	*ANN-P1 *	*ANN-P2 *	*ANN-P3 *
Control (negative) sample set	28/30 (93.3%)	26/30 (86.7%)	27/30 (90.0%)
Positive sample set	23/30 (76.7%)	25/30 (83.3%)	25/30 (83.3%)

Overall	51/60 (85.0%)	51/60 (85.0%)	52/60 (86.7%)

**Table 5:** Correct classifications for the organophosphate group (number of samples and corresponding percentages) during the testing process of the ANNs.

	Correct classification
Control (negative) sample set	36/50 (72.0%)
Positive sample set	45/50 (90.0%)

Overall	81/100 (81.0%)

**Table 6:** Correct classifications for the carbamate group (number of samples and corresponding percentages) during the testing process of the ANNs.

	Correct classification
Control (negative) sample set	25/30 (83.3%)
Positive sample set	26/30 (86.7%)

Overall	51/60 (85.0%)

## Abbreviations

ANN: Artificial neural network
BERA: Bioelectric recognition assay
MRL: Maximum residue level
PBS: Phosphate buffer saline.
